# 3β-Hydroxy-28-norolea-12,17-dien-11-one

**DOI:** 10.1107/S1600536814014998

**Published:** 2014-07-02

**Authors:** Werner Seebacher, Robert Weis, Johanna Faist, Robert Saf, Ferdinand Belaj

**Affiliations:** a Institute of Pharmaceutical Sciences, Department of Pharmaceutical Chemistry, Karl-Franzens University Graz, Schubertstrasse 1, A-8010 Graz, Austria; b Institute of Chemical Technology of Organic Materials, Erzherzog-Johann University, Stremayrgasse 26/1, A-8010 Graz, Austria; c Institute of Chemistry, Karl-Franzens University Graz, Schubertstrasse 1, A-8010 Graz, Austria

**Keywords:** crystal structure

## Abstract

The title compound, C_29_H_44_O_2_, was formed by treatment of 11-oxooleanolic acid under strong alkaline conditions. The absolute structure of the chiral mol­ecules could not be determined reliably from the diffraction data, but is known from other triterpenes. The asymmetric unit consists of two mol­ecules, 1 and 2. In both mol­ecules, rings *A* and *B* show chair conformations. The other rings show mixed forms between envelope and half-chair conformations with atoms in positions 8, 15 and 21 forming the flaps in rings *C*, *D* and *E*, respectively. Rings *D* and *E* of mol­ecule 2 are disordered over two orientations, with occupancies of 0.557 (4) and 0.443 (4), which differ in the direction of the flap in ring *E*. In the crystal, mol­ecules 1, as well as the mol­ecules 2, are linked by O—H⋯O hydrogen bonds, forming chains parallel to the *b* axis.

## Related literature   

For the synthesis of 11-oxo oleanolic acid, see: Ruzicka *et al.* (1938 ▶).
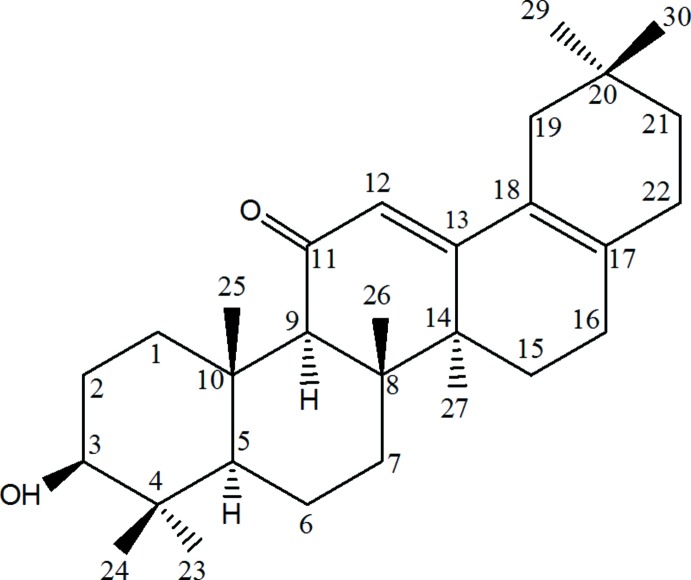


## Experimental   

### 

#### Crystal data  

C_29_H_44_O_2_
*M* *_r_* = 424.64
Monoclinic, 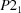

*a* = 12.2678 (5) Å
*b* = 16.0544 (6) Å
*c* = 12.9903 (5) Å
β = 104.448 (2)°
*V* = 2477.55 (17) Å^3^
*Z* = 4
Mo *K*α radiation
μ = 0.07 mm^−1^
*T* = 100 K
0.38 × 0.35 × 0.27 mm

#### Data collection  

Bruker APEXII CCD diffractometer
Absorption correction: multi-scan (*SADABS*; Bruker, 2010 ▶) *T*_min_ = 0.538, *T*_max_ = 0.745
15485 measured reflections
5293 independent reflections
4654 reflections with *I* > 2σ(*I*)
*R*_int_ = 0.031

#### Refinement  

*R*[*F* ^2^ > 2σ(*F* ^2^)] = 0.038
*wR*(*F* ^2^) = 0.103
*S* = 1.04
5293 reflections
685 parameters
31 restraints
H atoms treated by a mixture of independent and constrained refinement
Δρ_max_ = 0.22 e Å^−3^
Δρ_min_ = −0.18 e Å^−3^

### 

Data collection: *APEX2* (Bruker, 2010 ▶); cell refinement: *SAINT* (Bruker, 2010 ▶); data reduction: *SAINT*; program(s) used to solve structure: *SHELXS97* (Sheldrick, 2008 ▶); program(s) used to refine structure: *SHELXL97* (Sheldrick, 2008 ▶); molecular graphics: modified *ORTEP* (Johnson, 1965 ▶); software used to prepare material for publication: *SHELXL97*.

## Supplementary Material

Crystal structure: contains datablock(s) global, I. DOI: 10.1107/S1600536814014998/fy2111sup1.cif

Structure factors: contains datablock(s) I. DOI: 10.1107/S1600536814014998/fy2111Isup2.hkl

CCDC reference: 1010257

Additional supporting information:  crystallographic information; 3D view; checkCIF report

## Figures and Tables

**Table 1:** Hydrogen-bond geometry (Å, °)

*D*—H⋯*A*	*D*—H	H⋯*A*	*D*⋯*A*	*D*—H⋯*A*
O3—H3⋯O11^i^	0.84	2.04 (2)	2.792 (2)	148 (4)
O33—H33⋯O41^ii^	0.84	2.13 (2)	2.921 (2)	158 (4)

Symmetry codes: (i) 
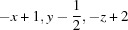
; (ii) 
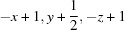
.

**Fig. 1.**
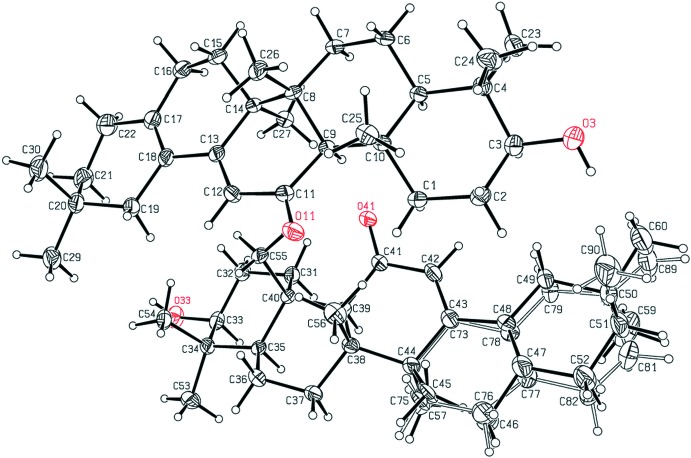
ORTEP plot (Johnson, 1965) of the asymmetric unit showing the atomic numbering scheme. The probability ellipsoids are drawn at the 50% probability level. The disordered part is drawn with open bonds for the fragment where the atoms have site occupation factors of 0.443 (4).

**Fig. 2.**
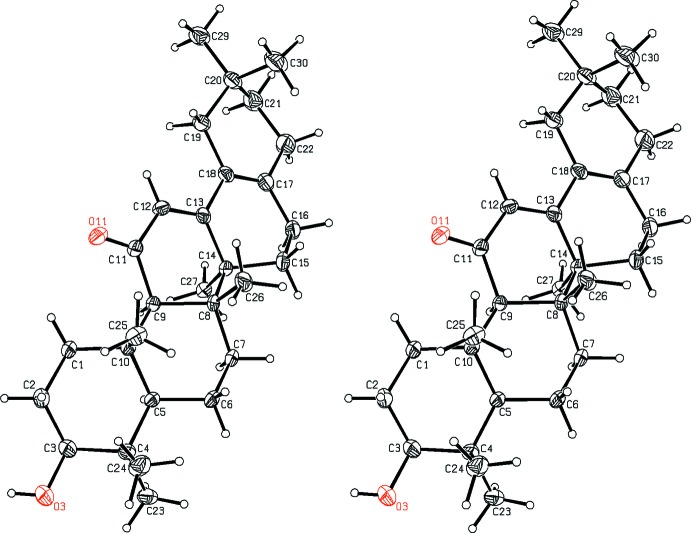
Stereoscopic ORTEP plot (Johnson, 1965) of molecule 1 showing the atomic numbering scheme. The probability ellipsoids are drawn at the 50% probability level.

**Fig. 3.**
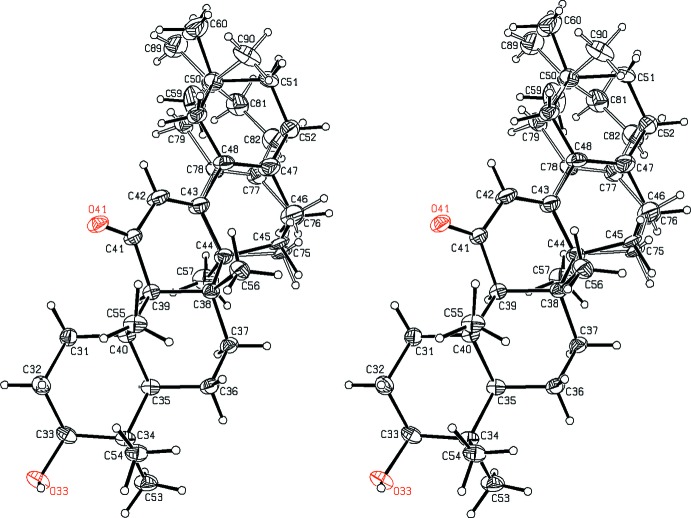
Stereoscopic ORTEP plot (Johnson, 1965) of molecule 2 showing the atomic numbering scheme. The probability ellipsoids are drawn at the 50% probability level. The disordered part is drawn with open bonds for the fragment where the atoms have site occupation factors of 0.443 (4).

## supplementary crystallographic information

### S1. Comment

Treatment of 11-oxo-oleanolic acid with potassium hydroxide in diethylene glycol at higher temperatures yielded the decarboxylation product 3β-hydroxy-28-norolea-12,17-dien-11-one under formation of a conjugated double bond system. This neutral compound crystallized from hexane in form of prisms. The asymmetric unit consists of two molecules 1 and 2 (Fig. 1). Rings D and E of molecule 2 are disordered over two orientations (Fig. 3). The molecules 1 as well as the molecules 2 are interconnected by hydrogen bonds to form chains parallel to the monoclinic *b* axis (Table 1).

### S2. Experimental

Potassium hydroxide (200 mg, 3.56 mmol) was added to 1.4 ml of diethylene glycol and stirred at 100°C. To the brown solution, 11-oxooleanolic acid (480 mg, 1.02 mmol) was added and heated up to 220°C. The reaction mixture was stirred under reflux for 4 h at this temperature. After cooling to room temperature, the reaction mixture was brought to pH = 1 (pH-paper) by addition of water and concentrated HCl. The precipitate was filtered by suction and washed with water. It was dissolved in chloroform and extracted twice with sodium hydroxide solution (2 N) and the organic phase was washed with water, dried over calcium chloride, filtered off and evaporated *in vacuo*. The residue was purified by use of CC over silica using ether as eluent. The product was recrystallized from hexane to give 180 mg (42%) of pale yellow prisms. Single crystals were obtained by slow evaporation of the solvent. *M.p.*: 197 – 200°C; Rf = 0.52 (ether). [α]_D_^20^ = +188.4°; [α]_546_^20^ = +236.4°; (c = 0.154, CH_3_OH).

IR (KBr): ν = 3477 (*m*), 2953 (*s*), 2869 (*s*), 1648 (*s*), 1622 (*s*), 1590 (*m*), 1457 (w), 1386 (*m*), 1365 (w), 1323 (w), 1201 (w) cm^-1^; UV (EtOH): λ (log ε) = 297 (4.817), 206 (4.327) nm.

^1^H NMR (400 MHz, CDCl_3_, 24°C, in p.p.m.): δ 0.69 (d, J = 11.7 Hz, 1H, 5-H), 0.78 (s, 3H, 24-H), 0.87 (s, 6H, 29-H, 30-H), 0.91–0.94 (m, 1H, 1-H), 0.97 (s, 3H, 23-H), 1.02 (s, 3H, 26-H), 1.14 (s, 3H, 27-H), 1.15 (s, 3H, 25-H), 1.27–1.78 (m, 11H, 2-H, 6-H, 7-H, 15-H, 19-H, 21-H), 1.94–2.24 (m, 5H, 16-H, 19-H, 22-H), 2.38 (s, 1H, 9-H), 2.76 (dt, J = 13.3, 3.2 Hz, 1H, 1-H), 3.20 (dd, J = 11.0, 5.0 Hz, 1H, 3-H), 5.68 (s, 1H, 12-H).

^13^C NMR (100 MHz, CDCl_3_, 24°C, in p.p.m.): δ 15.61 (C-24), 16.81 (C-25), 17.59 (C-6), 18.14 (C-26), 18.46 (C-27), 26.43 (C-15), 27.31 (C-2), 28.03, 28.63 (C-29, C-30), 28.07 (C-23), 28.54 (C-16), 29.23 (C-20), 29.91 (C-22), 33.82 (C-7), 34.57 (C-21), 37.13 (C-10), 38.99 (C-19), 39.08 (C-4), 39.12 (C-1), 42.24 (C-14), 43.64 (C-8), 55.23 (C-5), 60.91 (C-9), 78.76 (C-3), 119.99 (C-12), 125.33 (C-18), 141.64 (C-17), 158.40 (C-13), 200.76 (C-11).

MS (ES^+^): m/*z* (%) = 425 [MH^+^] (100.0), 317 (2.0), 143 (3.9), 130 (10.5), 120 (12.5), 115 (33.6); C_29_H_44_O_2_ (424.67). HRMS (MALDI): calcd. for (C_29_H_45_O_2_) [MH^+^]: 425.3420; found: 425.3469.

All NMR data were recorded using a Varian UnityInova spectrometer 400 MHz; TMS was used as internal standard. For optical rotation measurements a 241 MC polarimeter (Perkin-Elmer) was used. A Varian MAT 711 mass spectrometer was used with 70 eV electron ionization (EI) and field desorption. HRMS was performed on a Micromass Tofspec. IR spectra were measured with a System 2000 FTIR spectrometer (Perkin-Elmer) and UV-visible spectra with a Lambda 17 spectrophotometer (Perkin-Elmer).

### S3. Refinement

Due to the absence of heavier elements the absolute structure of the chiral molecules could not be determined reliably from the data but is known from other triterpenes. The symmetry-equivalent reflections including 3538 Friedel pairs were averaged.

The asymmetric unit consists of two molecules (1, 2). In molecule 2 rings D and E are disordered over two orientations and were refined with site occupation factors of 0.557 (4) and 0.443 (4), respectively. The same anisotropic displacement parameters were used for three atoms and 'rigid bond' restraints were applied for the atoms of the disordered part. The equivalent bonds in this disordered part were restrained to have the same lengths.

The other non-hydrogen atoms were refined with anisotropic displacement parameters without any constraints.

The O—H distances were fixed to 0.84 Å and the H atoms of the OH groups were refined with a common isotropic displacement parameter without any constraints to the bond angles. The H atoms of the tertiary C—H groups were refined with common isotropic displacement parameters and all *X*—C—H angles equal at a C—H distance of 1.00 Å. The H atoms of the CH_2_ groups were refined with common isotropic displacement parameters for the H atoms of the same group (or of the same ring in the disordered part, resp.) and idealized geometry with approximately tetrahedral angles and C—H distances of 0.99 Å. The H atoms at C12 and C42 were put at the external bisector of the C—C—C angle at a C—H distance of 0.95 Å. The H atoms of the methyl groups were refined with common isotropic displacement parameters for the H atoms of the same group (or for all the methyl groups in the disordered part, resp.) and idealized geometries with tetrahedral angles, enabling rotation around the C—C bond, and C—H distances of 0.98 Å.

### Crystal data

**Table:**

C_29_H_44_O_2_	*F*(000) = 936
*M**_r_* = 424.64	*D*_x_ = 1.138 Mg m^−^^3^
Monoclinic, *P*2_1_	Melting point = 470–473 K
Hall symbol: P 2yb	Mo *K*α radiation, λ = 0.71073 Å
*a* = 12.2678 (5) Å	Cell parameters from 7273 reflections
*b* = 16.0544 (6) Å	θ = 2.5–26.4°
*c* = 12.9903 (5) Å	µ = 0.07 mm^−^^1^
β = 104.448 (2)°	*T* = 100 K
*V* = 2477.55 (17) Å^3^	Block, pale yellow
*Z* = 4	0.38 × 0.35 × 0.27 mm

### Data collection

**Table:**

Bruker APEXII CCD diffractometer	5293 independent reflections
Radiation source: fine-focus sealed tube	4654 reflections with *I* > 2σ(*I*)
Graphite monochromator	*R*_int_ = 0.031
φ and ω scans	θ_max_ = 26.5°, θ_min_ = 2.4°
Absorption correction: multi-scan (*SADABS*; Bruker, 2010)	*h* = −15→15
*T*_min_ = 0.538, *T*_max_ = 0.745	*k* = −19→20
15485 measured reflections	*l* = −16→14

### Refinement

**Table:**

Refinement on *F*^2^	Primary atom site location: structure-invariant direct methods
Least-squares matrix: full	Secondary atom site location: difference Fourier map
*R*[*F*^2^ > 2σ(*F*^2^)] = 0.038	Hydrogen site location: inferred from neighbouring sites
*wR*(*F*^2^) = 0.103	H atoms treated by a mixture of independent and constrained refinement
*S* = 1.04	*w* = 1/[σ^2^(*F*_o_^2^) + (0.0597*P*)^2^ + 0.4012*P*] where *P* = (*F*_o_^2^ + 2*F*_c_^2^)/3
5293 reflections	(Δ/σ)_max_ = 0.001
685 parameters	Δρ_max_ = 0.22 e Å^−^^3^
31 restraints	Δρ_min_ = −0.18 e Å^−^^3^

### Special details

**Table:**

Geometry. All e.s.d.'s (except the e.s.d. in the dihedral angle between two l.s. planes) are estimated using the full covariance matrix. The cell e.s.d.'s are taken into account individually in the estimation of e.s.d.'s in distances, angles and torsion angles; correlations between e.s.d.'s in cell parameters are only used when they are defined by crystal symmetry. An approximate (isotropic) treatment of cell e.s.d.'s is used for estimating e.s.d.'s involving l.s. planes.
Refinement. Refinement of *F*^2^ against ALL reflections. The weighted *R*-factor *wR* and goodness of fit *S* are based on *F*^2^, conventional *R*-factors *R* are based on *F*, with *F* set to zero for negative *F*^2^. The threshold expression of *F*^2^ > σ(*F*^2^) is used only for calculating *R*-factors(gt) *etc*. and is not relevant to the choice of reflections for refinement. *R*-factors based on *F*^2^ are statistically about twice as large as those based on *F*, and *R*- factors based on ALL data will be even larger.

### Fractional atomic coordinates and isotropic or equivalent isotropic displacement parameters (Å^2^)

**Table:**

	*x*	*y*	*z*	*U*_iso_*/*U*_eq_	Occ. (<1)
C1	0.56673 (19)	0.70580 (14)	0.9397 (2)	0.0220 (5)	
H11	0.5212	0.7438	0.9722	0.021 (5)*	
H12	0.5384	0.7097	0.8615	0.021 (5)*	
C2	0.5519 (2)	0.61639 (15)	0.9748 (2)	0.0252 (5)	
H21	0.5742	0.6135	1.0534	0.029 (5)*	
H22	0.4714	0.6007	0.9511	0.029 (5)*	
C3	0.6218 (2)	0.55456 (15)	0.9297 (2)	0.0254 (5)	
H31	0.5922	0.5534	0.8507	0.020 (3)*	
O3	0.61486 (16)	0.47186 (12)	0.96916 (18)	0.0397 (5)	
H3	0.5463 (8)	0.460 (2)	0.960 (3)	0.063 (8)*	
C4	0.74744 (19)	0.57634 (14)	0.95482 (19)	0.0208 (5)	
C5	0.75942 (18)	0.66913 (14)	0.92399 (18)	0.0177 (4)	
H5	0.7260	0.6715	0.8455	0.020 (3)*	
C6	0.88191 (19)	0.69683 (15)	0.9390 (2)	0.0226 (5)	
H61	0.9164	0.7073	1.0153	0.030 (5)*	
H62	0.9253	0.6522	0.9147	0.030 (5)*	
C7	0.88569 (19)	0.77608 (14)	0.8752 (2)	0.0232 (5)	
H71	0.8529	0.7643	0.7990	0.029 (5)*	
H72	0.9652	0.7924	0.8836	0.029 (5)*	
C8	0.82157 (17)	0.84966 (14)	0.90925 (19)	0.0197 (5)	
C9	0.70127 (17)	0.81911 (13)	0.91449 (18)	0.0172 (4)	
H9	0.6611	0.8087	0.8386	0.020 (3)*	
C10	0.69188 (18)	0.73431 (14)	0.97192 (17)	0.0179 (4)	
C11	0.63892 (18)	0.89211 (15)	0.94712 (18)	0.0205 (5)	
O11	0.57411 (14)	0.88509 (11)	1.00575 (15)	0.0282 (4)	
C12	0.65632 (19)	0.97442 (14)	0.90521 (18)	0.0209 (5)	
H121	0.6089	1.0185	0.9163	0.020 (3)*	
C13	0.73478 (18)	0.99204 (14)	0.85198 (18)	0.0196 (5)	
C14	0.80878 (18)	0.92267 (14)	0.82496 (19)	0.0199 (5)	
C15	0.92351 (19)	0.96124 (15)	0.8247 (2)	0.0259 (5)	
H151	0.9596	0.9824	0.8967	0.026 (5)*	
H152	0.9729	0.9177	0.8068	0.026 (5)*	
C16	0.9116 (2)	1.03230 (15)	0.7450 (2)	0.0285 (6)	
H161	0.9854	1.0601	0.7537	0.036 (6)*	
H162	0.8888	1.0095	0.6720	0.036 (6)*	
C17	0.8259 (2)	1.09517 (15)	0.7596 (2)	0.0269 (5)	
C18	0.74776 (19)	1.07751 (14)	0.81372 (19)	0.0221 (5)	
C19	0.6686 (2)	1.14449 (14)	0.8350 (2)	0.0246 (5)	
H191	0.6638	1.1395	0.9097	0.039 (6)*	
H192	0.5925	1.1339	0.7889	0.039 (6)*	
C20	0.7025 (2)	1.23423 (15)	0.8162 (2)	0.0295 (6)	
C21	0.7361 (2)	1.23658 (17)	0.7106 (2)	0.0355 (6)	
H211	0.7563	1.2944	0.6963	0.051 (7)*	
H212	0.6708	1.2197	0.6526	0.051 (7)*	
C22	0.8354 (2)	1.17911 (17)	0.7106 (2)	0.0366 (6)	
H221	0.8407	1.1710	0.6365	0.048 (6)*	
H222	0.9057	1.2064	0.7504	0.048 (6)*	
C23	0.8009 (2)	0.51984 (15)	0.8847 (2)	0.0280 (5)	
H231	0.8830	0.5251	0.9068	0.038 (5)*	
H232	0.7740	0.5367	0.8102	0.038 (5)*	
H233	0.7796	0.4618	0.8926	0.038 (5)*	
C24	0.8059 (2)	0.55615 (17)	1.0711 (2)	0.0301 (6)	
H241	0.8103	0.4956	1.0808	0.038 (5)*	
H242	0.7626	0.5802	1.1178	0.038 (5)*	
H243	0.8820	0.5797	1.0888	0.038 (5)*	
C25	0.7305 (2)	0.74225 (15)	1.09450 (19)	0.0255 (5)	
H251	0.7163	0.7991	1.1155	0.031 (4)*	
H252	0.8111	0.7300	1.1183	0.031 (4)*	
H253	0.6884	0.7027	1.1272	0.031 (4)*	
C26	0.8895 (2)	0.88082 (16)	1.0190 (2)	0.0270 (5)	
H261	0.8883	0.8383	1.0729	0.039 (5)*	
H262	0.8558	0.9324	1.0371	0.039 (5)*	
H263	0.9674	0.8915	1.0165	0.039 (5)*	
C27	0.75315 (19)	0.89320 (15)	0.71071 (19)	0.0222 (5)	
H271	0.6876	0.8585	0.7113	0.030 (4)*	
H272	0.8075	0.8606	0.6834	0.030 (4)*	
H273	0.7291	0.9417	0.6649	0.030 (4)*	
C29	0.6023 (2)	1.29271 (16)	0.8107 (2)	0.0344 (6)	
H291	0.6256	1.3504	0.8035	0.039 (5)*	
H292	0.5763	1.2871	0.8758	0.039 (5)*	
H293	0.5410	1.2780	0.7492	0.039 (5)*	
C30	0.8008 (3)	1.26177 (18)	0.9081 (3)	0.0438 (8)	
H301	0.8598	1.2191	0.9209	0.051 (5)*	
H302	0.7740	1.2694	0.9725	0.051 (5)*	
H303	0.8315	1.3145	0.8897	0.051 (5)*	
C31	0.3809 (2)	0.97248 (14)	0.43848 (19)	0.0227 (5)	
H311	0.4442	0.9347	0.4366	0.029 (5)*	
H312	0.3126	0.9506	0.3881	0.029 (5)*	
C32	0.4067 (2)	1.05953 (15)	0.40242 (19)	0.0243 (5)	
H321	0.4782	1.0797	0.4495	0.030 (5)*	
H322	0.4168	1.0562	0.3292	0.030 (5)*	
C33	0.3134 (2)	1.12142 (14)	0.40473 (19)	0.0232 (5)	
H331	0.2435	1.0986	0.3559	0.031 (4)*	
O33	0.33349 (16)	1.19994 (10)	0.36145 (14)	0.0300 (4)	
H33	0.387 (2)	1.225 (2)	0.402 (2)	0.063 (8)*	
C34	0.28831 (19)	1.12850 (14)	0.51480 (19)	0.0204 (5)	
C35	0.26916 (17)	1.03865 (13)	0.55319 (18)	0.0181 (4)	
H35	0.2014	1.0179	0.4994	0.031 (4)*	
C36	0.2348 (2)	1.03607 (14)	0.65826 (19)	0.0228 (5)	
H361	0.3018	1.0450	0.7180	0.031 (5)*	
H362	0.1802	1.0812	0.6598	0.031 (5)*	
C37	0.1818 (2)	0.95215 (15)	0.6708 (2)	0.0228 (5)	
H371	0.1132	0.9451	0.6123	0.033 (5)*	
H372	0.1585	0.9520	0.7385	0.033 (5)*	
C38	0.26091 (18)	0.87747 (14)	0.67029 (18)	0.0196 (5)	
C39	0.31363 (17)	0.88578 (14)	0.57250 (17)	0.0176 (4)	
H39	0.2492	0.8762	0.5092	0.031 (4)*	
C40	0.36224 (17)	0.97260 (14)	0.55178 (17)	0.0174 (4)	
C41	0.39175 (19)	0.81162 (14)	0.57326 (19)	0.0222 (5)	
O41	0.48323 (14)	0.81579 (10)	0.55101 (15)	0.0273 (4)	
C42	0.3518 (2)	0.73038 (15)	0.6013 (2)	0.0329 (6)	
H421	0.3924	0.6822	0.5903	0.031 (4)*	
C43	0.2621 (2)	0.71939 (15)	0.6414 (2)	0.0306 (6)	
C44	0.1919 (2)	0.79335 (15)	0.6609 (2)	0.0251 (5)	
C45	0.161 (2)	0.7699 (11)	0.764 (2)	0.032 (2)	0.557 (4)
H451	0.2311	0.7597	0.8192	0.036 (6)*	0.557 (4)
H452	0.1229	0.8183	0.7872	0.036 (6)*	0.557 (4)
C46	0.0848 (17)	0.6943 (10)	0.758 (2)	0.0439 (19)	0.557 (4)
H461	0.0180	0.6975	0.6974	0.036 (6)*	0.557 (4)
H462	0.0603	0.6869	0.8250	0.036 (6)*	0.557 (4)
C47	0.1661 (7)	0.6252 (6)	0.7437 (7)	0.0365 (18)	0.557 (4)
C48	0.2400 (7)	0.6352 (6)	0.6817 (7)	0.0259 (16)	0.557 (4)
C49	0.3009 (5)	0.5626 (4)	0.6522 (5)	0.0278 (12)	0.557 (4)
H491	0.3782	0.5622	0.6989	0.062 (7)*	0.557 (4)
H492	0.3073	0.5707	0.5784	0.062 (7)*	0.557 (4)
C50	0.247 (2)	0.476 (3)	0.6595 (16)	0.0299 (9)	0.557 (4)
C51	0.2104 (4)	0.4726 (3)	0.7625 (4)	0.0316 (11)	0.557 (4)
H511	0.1750	0.4179	0.7676	0.062 (7)*	0.557 (4)
H512	0.2776	0.4772	0.8230	0.062 (7)*	0.557 (4)
C52	0.1282 (6)	0.5408 (4)	0.7713 (6)	0.0375 (14)	0.557 (4)
H521	0.0539	0.5280	0.7232	0.062 (7)*	0.557 (4)
H522	0.1191	0.5422	0.8449	0.062 (7)*	0.557 (4)
C53	0.1790 (2)	1.17875 (16)	0.5008 (2)	0.0306 (6)	
H531	0.1842	1.2300	0.4615	0.033 (4)*	
H532	0.1676	1.1928	0.5708	0.033 (4)*	
H533	0.1153	1.1454	0.4613	0.033 (4)*	
C54	0.3818 (2)	1.17710 (15)	0.5922 (2)	0.0249 (5)	
H541	0.3797	1.2356	0.5701	0.036 (4)*	
H542	0.4551	1.1531	0.5919	0.036 (4)*	
H543	0.3703	1.1737	0.6641	0.036 (4)*	
C55	0.47564 (18)	0.99181 (15)	0.6325 (2)	0.0237 (5)	
H551	0.5144	0.9395	0.6576	0.029 (4)*	
H552	0.4614	1.0225	0.6931	0.029 (4)*	
H553	0.5228	1.0256	0.5980	0.029 (4)*	
C56	0.3544 (2)	0.87866 (16)	0.77538 (19)	0.0287 (5)	
H561	0.4030	0.9272	0.7761	0.041 (5)*	
H562	0.3993	0.8276	0.7809	0.041 (5)*	
H563	0.3199	0.8819	0.8357	0.041 (5)*	
C57	0.0847 (2)	0.79513 (17)	0.5679 (2)	0.0371 (7)	
H571	0.0564	0.7383	0.5521	0.047 (5)*	
H572	0.1029	0.8191	0.5048	0.047 (5)*	
H573	0.0269	0.8292	0.5878	0.047 (5)*	
C59	0.1462 (6)	0.4640 (4)	0.5643 (5)	0.0533 (17)	0.557 (4)
H591	0.1723	0.4599	0.4991	0.066 (6)*	0.557 (4)
H592	0.0950	0.5116	0.5589	0.066 (6)*	0.557 (4)
H593	0.1064	0.4128	0.5739	0.066 (6)*	0.557 (4)
C60	0.3364 (7)	0.4090 (4)	0.6623 (8)	0.057 (2)	0.557 (4)
H601	0.4007	0.4188	0.7231	0.066 (6)*	0.557 (4)
H602	0.3615	0.4111	0.5964	0.066 (6)*	0.557 (4)
H603	0.3040	0.3541	0.6692	0.066 (6)*	0.557 (4)
C75	0.151 (3)	0.7840 (15)	0.765 (3)	0.032 (2)	0.443 (4)
H751	0.2144	0.7912	0.8284	0.036 (6)*	0.443 (4)
H752	0.0929	0.8266	0.7675	0.036 (6)*	0.443 (4)
C76	0.101 (2)	0.6971 (13)	0.764 (3)	0.0439 (19)	0.443 (4)
H761	0.0183	0.7037	0.7393	0.036 (6)*	0.443 (4)
H762	0.1167	0.6789	0.8390	0.036 (6)*	0.443 (4)
C77	0.1322 (8)	0.6236 (7)	0.7014 (8)	0.0305 (19)	0.443 (4)
C78	0.2109 (9)	0.6345 (8)	0.6472 (7)	0.0195 (16)	0.443 (4)
C79	0.2649 (7)	0.5606 (5)	0.6046 (6)	0.0277 (15)	0.443 (4)
H791	0.2351	0.5579	0.5265	0.062 (7)*	0.443 (4)
H792	0.3471	0.5700	0.6195	0.062 (7)*	0.443 (4)
C80	0.243 (3)	0.478 (4)	0.653 (2)	0.0299 (9)	0.443 (4)
C81	0.1184 (6)	0.4708 (4)	0.6487 (6)	0.0394 (15)	0.443 (4)
H811	0.0737	0.4752	0.5741	0.062 (7)*	0.443 (4)
H812	0.1029	0.4158	0.6764	0.062 (7)*	0.443 (4)
C82	0.0838 (7)	0.5386 (5)	0.7140 (8)	0.0408 (18)	0.443 (4)
H821	0.1072	0.5225	0.7899	0.062 (7)*	0.443 (4)
H822	0.0006	0.5427	0.6941	0.062 (7)*	0.443 (4)
C89	0.2759 (8)	0.4065 (4)	0.5871 (8)	0.051 (2)	0.443 (4)
H891	0.2724	0.3533	0.6232	0.066 (6)*	0.443 (4)
H892	0.3525	0.4155	0.5796	0.066 (6)*	0.443 (4)
H893	0.2234	0.4053	0.5165	0.066 (6)*	0.443 (4)
C90	0.3167 (7)	0.4700 (5)	0.7668 (6)	0.0491 (19)	0.443 (4)
H901	0.3058	0.5193	0.8076	0.066 (6)*	0.443 (4)
H902	0.3961	0.4660	0.7655	0.066 (6)*	0.443 (4)
H903	0.2951	0.4200	0.8001	0.066 (6)*	0.443 (4)

### Atomic displacement parameters (Å^2^)

**Table:**

	*U*^11^	*U*^22^	*U*^33^	*U*^12^	*U*^13^	*U*^23^
C1	0.0153 (11)	0.0214 (11)	0.0293 (13)	0.0018 (9)	0.0057 (9)	0.0022 (9)
C2	0.0197 (11)	0.0241 (12)	0.0332 (14)	−0.0016 (10)	0.0091 (10)	0.0048 (10)
C3	0.0245 (12)	0.0204 (11)	0.0318 (13)	−0.0014 (10)	0.0078 (10)	0.0043 (10)
O3	0.0298 (10)	0.0244 (10)	0.0685 (14)	−0.0020 (8)	0.0190 (10)	0.0115 (9)
C4	0.0209 (11)	0.0174 (11)	0.0254 (12)	0.0030 (9)	0.0082 (9)	0.0060 (9)
C5	0.0149 (10)	0.0173 (10)	0.0209 (11)	0.0022 (8)	0.0047 (8)	0.0017 (9)
C6	0.0134 (10)	0.0214 (11)	0.0322 (13)	0.0043 (9)	0.0043 (9)	0.0041 (10)
C7	0.0134 (10)	0.0218 (11)	0.0355 (14)	0.0013 (9)	0.0079 (9)	0.0013 (10)
C8	0.0093 (9)	0.0196 (11)	0.0275 (12)	−0.0006 (8)	−0.0004 (9)	−0.0018 (9)
C9	0.0128 (10)	0.0169 (10)	0.0202 (11)	0.0005 (8)	0.0011 (8)	−0.0012 (8)
C10	0.0143 (10)	0.0188 (10)	0.0199 (11)	0.0015 (8)	0.0032 (8)	0.0011 (9)
C11	0.0160 (10)	0.0206 (11)	0.0240 (11)	0.0010 (9)	0.0032 (9)	−0.0016 (9)
O11	0.0253 (8)	0.0237 (8)	0.0396 (10)	0.0047 (7)	0.0159 (8)	0.0006 (8)
C12	0.0195 (10)	0.0174 (11)	0.0247 (12)	0.0038 (9)	0.0034 (9)	−0.0018 (9)
C13	0.0146 (10)	0.0182 (11)	0.0220 (11)	−0.0013 (9)	−0.0028 (8)	−0.0017 (9)
C14	0.0128 (10)	0.0187 (10)	0.0268 (12)	−0.0003 (9)	0.0026 (9)	−0.0039 (9)
C15	0.0140 (10)	0.0244 (12)	0.0374 (14)	−0.0017 (9)	0.0032 (10)	0.0043 (11)
C16	0.0188 (11)	0.0237 (12)	0.0418 (15)	−0.0028 (10)	0.0055 (10)	0.0049 (11)
C17	0.0222 (12)	0.0209 (11)	0.0352 (14)	−0.0024 (10)	0.0025 (10)	0.0011 (10)
C18	0.0190 (11)	0.0176 (11)	0.0258 (12)	−0.0023 (9)	−0.0017 (9)	−0.0021 (9)
C19	0.0226 (11)	0.0186 (11)	0.0295 (13)	−0.0001 (9)	0.0007 (10)	−0.0021 (10)
C20	0.0239 (12)	0.0182 (11)	0.0395 (15)	−0.0006 (10)	−0.0051 (11)	−0.0029 (10)
C21	0.0318 (14)	0.0232 (13)	0.0480 (17)	−0.0018 (11)	0.0033 (12)	0.0082 (12)
C22	0.0328 (14)	0.0273 (13)	0.0506 (18)	−0.0017 (12)	0.0119 (13)	0.0079 (12)
C23	0.0313 (13)	0.0181 (11)	0.0371 (15)	0.0031 (10)	0.0131 (11)	0.0030 (10)
C24	0.0280 (12)	0.0301 (13)	0.0323 (14)	0.0075 (11)	0.0080 (10)	0.0108 (11)
C25	0.0252 (12)	0.0266 (12)	0.0239 (12)	0.0048 (10)	0.0047 (10)	−0.0015 (10)
C26	0.0195 (11)	0.0234 (12)	0.0322 (13)	−0.0024 (10)	−0.0043 (10)	−0.0002 (10)
C27	0.0194 (11)	0.0206 (11)	0.0267 (12)	0.0009 (9)	0.0057 (9)	−0.0020 (9)
C29	0.0310 (14)	0.0204 (12)	0.0451 (16)	0.0021 (11)	−0.0030 (12)	−0.0006 (11)
C30	0.0352 (15)	0.0260 (13)	0.058 (2)	−0.0010 (12)	−0.0126 (14)	−0.0082 (13)
C31	0.0256 (11)	0.0195 (11)	0.0245 (12)	−0.0029 (10)	0.0090 (9)	−0.0028 (9)
C32	0.0255 (12)	0.0222 (11)	0.0261 (12)	−0.0029 (10)	0.0079 (10)	0.0014 (10)
C33	0.0228 (11)	0.0194 (11)	0.0242 (12)	−0.0043 (9)	−0.0002 (9)	0.0030 (9)
O33	0.0358 (10)	0.0218 (9)	0.0288 (10)	−0.0029 (8)	0.0014 (8)	0.0079 (7)
C34	0.0181 (10)	0.0157 (10)	0.0251 (12)	0.0013 (9)	0.0011 (9)	0.0008 (9)
C35	0.0125 (10)	0.0176 (11)	0.0212 (11)	−0.0011 (8)	−0.0014 (8)	−0.0008 (8)
C36	0.0238 (12)	0.0189 (11)	0.0261 (13)	0.0045 (9)	0.0069 (10)	−0.0007 (9)
C37	0.0209 (11)	0.0205 (11)	0.0292 (13)	0.0036 (9)	0.0106 (10)	0.0009 (10)
C38	0.0172 (10)	0.0193 (11)	0.0221 (12)	0.0030 (9)	0.0045 (9)	0.0025 (9)
C39	0.0138 (10)	0.0179 (10)	0.0200 (11)	−0.0005 (9)	0.0021 (8)	−0.0012 (9)
C40	0.0139 (10)	0.0163 (10)	0.0206 (11)	−0.0009 (8)	0.0015 (8)	−0.0022 (9)
C41	0.0212 (11)	0.0179 (11)	0.0290 (13)	0.0015 (9)	0.0094 (10)	−0.0011 (9)
O41	0.0216 (8)	0.0195 (8)	0.0447 (11)	0.0025 (7)	0.0158 (8)	0.0011 (7)
C42	0.0335 (14)	0.0166 (12)	0.0562 (18)	0.0057 (10)	0.0256 (13)	0.0018 (11)
C43	0.0301 (14)	0.0192 (12)	0.0485 (16)	0.0022 (10)	0.0213 (12)	0.0025 (11)
C44	0.0236 (12)	0.0183 (11)	0.0372 (14)	0.0018 (10)	0.0146 (11)	0.0036 (10)
C45	0.043 (4)	0.014 (6)	0.0527 (18)	0.012 (4)	0.034 (3)	0.005 (4)
C46	0.047 (5)	0.0273 (16)	0.073 (3)	0.002 (2)	0.044 (3)	0.0098 (16)
C47	0.040 (5)	0.023 (3)	0.054 (5)	0.004 (3)	0.026 (4)	0.006 (4)
C48	0.019 (4)	0.020 (2)	0.039 (5)	0.005 (3)	0.007 (3)	0.006 (4)
C49	0.030 (3)	0.017 (2)	0.040 (4)	0.000 (2)	0.016 (3)	0.000 (3)
C50	0.0395 (19)	0.0182 (16)	0.035 (2)	−0.0023 (14)	0.0144 (13)	0.003 (3)
C51	0.043 (3)	0.018 (2)	0.037 (3)	0.0040 (19)	0.015 (2)	0.0093 (19)
C52	0.049 (4)	0.026 (3)	0.047 (4)	0.006 (3)	0.030 (3)	0.009 (3)
C53	0.0240 (12)	0.0239 (12)	0.0433 (15)	0.0070 (10)	0.0071 (11)	0.0065 (11)
C54	0.0265 (12)	0.0172 (11)	0.0283 (13)	−0.0024 (10)	0.0016 (10)	−0.0021 (9)
C55	0.0138 (10)	0.0211 (11)	0.0323 (13)	−0.0009 (9)	−0.0013 (9)	0.0008 (9)
C56	0.0313 (13)	0.0297 (13)	0.0232 (13)	0.0045 (11)	0.0032 (10)	0.0036 (10)
C57	0.0231 (12)	0.0283 (14)	0.0588 (19)	−0.0108 (11)	0.0082 (12)	0.0026 (13)
C59	0.066 (4)	0.059 (4)	0.034 (3)	−0.029 (3)	0.009 (2)	−0.012 (3)
C60	0.073 (5)	0.025 (3)	0.091 (6)	0.009 (3)	0.054 (5)	0.006 (3)
C75	0.043 (4)	0.014 (6)	0.0527 (18)	0.012 (4)	0.034 (3)	0.005 (4)
C76	0.047 (5)	0.0273 (16)	0.073 (3)	0.002 (2)	0.044 (3)	0.0098 (16)
C77	0.031 (5)	0.024 (3)	0.040 (6)	−0.001 (4)	0.015 (4)	0.004 (4)
C78	0.015 (5)	0.022 (3)	0.018 (5)	0.001 (3)	−0.003 (3)	0.003 (4)
C79	0.038 (5)	0.019 (3)	0.027 (4)	−0.004 (3)	0.009 (3)	0.001 (3)
C80	0.0395 (19)	0.0182 (16)	0.035 (2)	−0.0023 (14)	0.0144 (13)	0.003 (3)
C81	0.046 (3)	0.028 (3)	0.045 (4)	−0.013 (3)	0.014 (3)	0.002 (3)
C82	0.046 (5)	0.031 (3)	0.051 (5)	−0.012 (3)	0.022 (4)	0.002 (4)
C89	0.072 (6)	0.021 (3)	0.070 (6)	−0.002 (4)	0.040 (5)	0.000 (3)
C90	0.059 (5)	0.038 (4)	0.045 (4)	0.005 (3)	0.002 (3)	0.019 (3)

### Geometric parameters (Å, º)

**Table:**

C1—C2	1.531 (3)	C35—C36	1.526 (3)
C1—C10	1.556 (3)	C35—C40	1.562 (3)
C1—H11	0.99	C35—H35	1.00
C1—H12	0.99	C36—C37	1.522 (3)
C2—C3	1.522 (3)	C36—H361	0.99
C2—H21	0.99	C36—H362	0.99
C2—H22	0.99	C37—C38	1.544 (3)
C3—O3	1.433 (3)	C37—H371	0.99
C3—C4	1.534 (3)	C37—H372	0.99
C3—H31	1.00	C38—C56	1.548 (3)
O3—H3	0.84	C38—C39	1.568 (3)
C4—C24	1.536 (3)	C38—C44	1.583 (3)
C4—C23	1.543 (3)	C39—C41	1.527 (3)
C4—C5	1.559 (3)	C39—C40	1.566 (3)
C5—C6	1.532 (3)	C39—H39	1.00
C5—C10	1.558 (3)	C40—C55	1.550 (3)
C5—H5	1.00	C41—O41	1.229 (3)
C6—C7	1.525 (3)	C41—C42	1.471 (3)
C6—H61	0.99	C42—C43	1.341 (3)
C6—H62	0.99	C42—H421	0.95
C7—C8	1.544 (3)	C43—C48	1.498 (10)
C7—H71	0.99	C43—C78	1.510 (13)
C7—H72	0.99	C43—C44	1.525 (3)
C8—C26	1.544 (3)	C44—C45	1.53 (3)
C8—C9	1.573 (3)	C44—C57	1.548 (4)
C8—C14	1.585 (3)	C44—C75	1.57 (4)
C9—C11	1.517 (3)	C45—C46	1.521 (6)
C9—C10	1.571 (3)	C45—H451	0.99
C9—H9	1.00	C45—H452	0.99
C10—C25	1.549 (3)	C46—C47	1.535 (8)
C11—O11	1.236 (3)	C46—H461	0.99
C11—C12	1.465 (3)	C46—H462	0.99
C12—C13	1.348 (3)	C47—C48	1.365 (13)
C12—H121	0.95	C47—C52	1.504 (11)
C13—C18	1.481 (3)	C48—C49	1.485 (12)
C13—C14	1.532 (3)	C49—C50	1.55 (6)
C14—C15	1.538 (3)	C49—H491	0.99
C14—C27	1.545 (3)	C49—H492	0.99
C15—C16	1.523 (3)	C50—C51	1.52 (4)
C15—H151	0.99	C50—C59	1.529 (12)
C15—H152	0.99	C50—C60	1.530 (12)
C16—C17	1.504 (3)	C51—C52	1.512 (7)
C16—H161	0.99	C51—H511	0.99
C16—H162	0.99	C51—H512	0.99
C17—C18	1.352 (4)	C52—H521	0.99
C17—C22	1.507 (4)	C52—H522	0.99
C18—C19	1.520 (3)	C53—H531	0.98
C19—C20	1.536 (3)	C53—H532	0.98
C19—H191	0.99	C53—H533	0.98
C19—H192	0.99	C54—H541	0.98
C20—C21	1.528 (4)	C54—H542	0.98
C20—C29	1.534 (3)	C54—H543	0.98
C20—C30	1.536 (4)	C55—H551	0.98
C21—C22	1.529 (4)	C55—H552	0.98
C21—H211	0.99	C55—H553	0.98
C21—H212	0.99	C56—H561	0.98
C22—H221	0.99	C56—H562	0.98
C22—H222	0.99	C56—H563	0.98
C23—H231	0.98	C57—H571	0.98
C23—H232	0.98	C57—H572	0.98
C23—H233	0.98	C57—H573	0.98
C24—H241	0.98	C59—H591	0.98
C24—H242	0.98	C59—H592	0.98
C24—H243	0.98	C59—H593	0.98
C25—H251	0.98	C60—H601	0.98
C25—H252	0.98	C60—H602	0.98
C25—H253	0.98	C60—H603	0.98
C26—H261	0.98	C75—C76	1.521 (6)
C26—H262	0.98	C75—H751	0.99
C26—H263	0.98	C75—H752	0.99
C27—H271	0.98	C76—C77	1.535 (8)
C27—H272	0.98	C76—H761	0.99
C27—H273	0.98	C76—H762	0.99
C29—H291	0.98	C77—C78	1.341 (17)
C29—H292	0.98	C77—C82	1.513 (14)
C29—H293	0.98	C78—C79	1.527 (16)
C30—H301	0.98	C79—C80	1.52 (7)
C30—H302	0.98	C79—H791	0.99
C30—H303	0.98	C79—H792	0.99
C31—C32	1.532 (3)	C80—C81	1.53 (4)
C31—C40	1.545 (3)	C80—C90	1.534 (16)
C31—H311	0.99	C80—C89	1.534 (16)
C31—H312	0.99	C81—C82	1.505 (11)
C32—C33	1.522 (3)	C81—H811	0.99
C32—H321	0.99	C81—H812	0.99
C32—H322	0.99	C82—H821	0.99
C33—O33	1.427 (3)	C82—H822	0.99
C33—C34	1.540 (3)	C89—H891	0.98
C33—H331	1.00	C89—H892	0.98
O33—H33	0.84	C89—H893	0.98
C34—C54	1.536 (3)	C90—H901	0.98
C34—C53	1.537 (3)	C90—H902	0.98
C34—C35	1.563 (3)	C90—H903	0.98
			
C2—C1—C10	112.23 (19)	C33—C34—C35	107.99 (18)
C2—C1—H11	109.2	C36—C35—C40	110.97 (18)
C10—C1—H11	109.2	C36—C35—C34	114.04 (18)
C2—C1—H12	109.2	C40—C35—C34	117.02 (17)
C10—C1—H12	109.2	C36—C35—H35	104.4
H11—C1—H12	107.9	C40—C35—H35	104.4
C3—C2—C1	112.11 (19)	C34—C35—H35	104.4
C3—C2—H21	109.2	C37—C36—C35	109.91 (19)
C1—C2—H21	109.2	C37—C36—H361	109.7
C3—C2—H22	109.2	C35—C36—H361	109.7
C1—C2—H22	109.2	C37—C36—H362	109.7
H21—C2—H22	107.9	C35—C36—H362	109.7
O3—C3—C2	112.1 (2)	H361—C36—H362	108.2
O3—C3—C4	106.37 (19)	C36—C37—C38	113.62 (18)
C2—C3—C4	113.7 (2)	C36—C37—H371	108.8
O3—C3—H31	108.1	C38—C37—H371	108.8
C2—C3—H31	108.1	C36—C37—H372	108.8
C4—C3—H31	108.1	C38—C37—H372	108.8
C3—O3—H3	107 (3)	H371—C37—H372	107.7
C3—C4—C24	111.00 (19)	C37—C38—C56	108.34 (19)
C3—C4—C23	107.0 (2)	C37—C38—C39	108.93 (18)
C24—C4—C23	107.08 (19)	C56—C38—C39	110.42 (18)
C3—C4—C5	108.51 (18)	C37—C38—C44	109.75 (18)
C24—C4—C5	113.8 (2)	C56—C38—C44	110.06 (19)
C23—C4—C5	109.21 (18)	C39—C38—C44	109.32 (18)
C6—C5—C10	110.90 (18)	C41—C39—C40	115.46 (17)
C6—C5—C4	113.39 (18)	C41—C39—C38	108.27 (18)
C10—C5—C4	116.46 (18)	C40—C39—C38	117.82 (17)
C6—C5—H5	104.9	C41—C39—H39	104.6
C10—C5—H5	104.9	C40—C39—H39	104.6
C4—C5—H5	104.9	C38—C39—H39	104.6
C7—C6—C5	109.55 (19)	C31—C40—C55	108.89 (18)
C7—C6—H61	109.8	C31—C40—C35	107.13 (18)
C5—C6—H61	109.8	C55—C40—C35	113.10 (18)
C7—C6—H62	109.8	C31—C40—C39	108.42 (17)
C5—C6—H62	109.8	C55—C40—C39	112.14 (18)
H61—C6—H62	108.2	C35—C40—C39	106.95 (16)
C6—C7—C8	113.32 (19)	O41—C41—C42	119.2 (2)
C6—C7—H71	108.9	O41—C41—C39	124.4 (2)
C8—C7—H71	108.9	C42—C41—C39	116.45 (19)
C6—C7—H72	108.9	C43—C42—C41	124.7 (2)
C8—C7—H72	108.9	C43—C42—H421	117.6
H71—C7—H72	107.7	C41—C42—H421	117.6
C26—C8—C7	108.43 (19)	C42—C43—C48	119.4 (4)
C26—C8—C9	110.93 (19)	C42—C43—C78	122.0 (5)
C7—C8—C9	108.94 (18)	C42—C43—C44	120.9 (2)
C26—C8—C14	109.62 (19)	C48—C43—C44	119.1 (4)
C7—C8—C14	109.73 (19)	C78—C43—C44	115.8 (5)
C9—C8—C14	109.17 (17)	C43—C44—C45	103.2 (8)
C11—C9—C10	115.80 (18)	C43—C44—C57	106.8 (2)
C11—C9—C8	108.38 (18)	C45—C44—C57	109.8 (10)
C10—C9—C8	118.15 (17)	C43—C44—C75	112.7 (10)
C11—C9—H9	104.3	C57—C44—C75	106.4 (13)
C10—C9—H9	104.3	C43—C44—C38	111.34 (18)
C8—C9—H9	104.3	C45—C44—C38	112.8 (8)
C25—C10—C1	109.20 (18)	C57—C44—C38	112.2 (2)
C25—C10—C5	114.17 (18)	C75—C44—C38	107.3 (10)
C1—C10—C5	106.40 (18)	C46—C45—C44	116 (2)
C25—C10—C9	112.15 (18)	C46—C45—H451	108.3
C1—C10—C9	108.15 (17)	C44—C45—H451	108.3
C5—C10—C9	106.45 (17)	C46—C45—H452	108.3
O11—C11—C12	119.2 (2)	C44—C45—H452	108.3
O11—C11—C9	123.3 (2)	H451—C45—H452	107.4
C12—C11—C9	117.56 (19)	C45—C46—C47	100.0 (13)
C13—C12—C11	124.7 (2)	C45—C46—H461	111.8
C13—C12—H121	117.7	C47—C46—H461	111.8
C11—C12—H121	117.7	C45—C46—H462	111.8
C12—C13—C18	121.1 (2)	C47—C46—H462	111.8
C12—C13—C14	120.3 (2)	H461—C46—H462	109.5
C18—C13—C14	118.49 (19)	C48—C47—C52	122.5 (8)
C13—C14—C15	107.62 (18)	C48—C47—C46	122.5 (12)
C13—C14—C27	107.32 (18)	C52—C47—C46	111.8 (10)
C15—C14—C27	107.84 (19)	C47—C48—C49	120.7 (8)
C13—C14—C8	109.97 (18)	C47—C48—C43	121.1 (8)
C15—C14—C8	111.49 (18)	C49—C48—C43	118.2 (7)
C27—C14—C8	112.40 (18)	C48—C49—C50	116.0 (8)
C16—C15—C14	111.74 (19)	C48—C49—H491	108.3
C16—C15—H151	109.3	C50—C49—H491	108.3
C14—C15—H151	109.3	C48—C49—H492	108.3
C16—C15—H152	109.3	C50—C49—H492	108.3
C14—C15—H152	109.3	H491—C49—H492	107.4
H151—C15—H152	107.9	C51—C50—C59	110 (2)
C17—C16—C15	111.6 (2)	C51—C50—C60	109 (2)
C17—C16—H161	109.3	C59—C50—C60	111.1 (13)
C15—C16—H161	109.3	C51—C50—C49	108.2 (13)
C17—C16—H162	109.3	C59—C50—C49	110 (2)
C15—C16—H162	109.3	C60—C50—C49	109 (2)
H161—C16—H162	108.0	C52—C51—C50	113.0 (11)
C18—C17—C16	122.2 (2)	C52—C51—H511	109.0
C18—C17—C22	123.1 (2)	C50—C51—H511	109.0
C16—C17—C22	114.6 (2)	C52—C51—H512	109.0
C17—C18—C13	121.2 (2)	C50—C51—H512	109.0
C17—C18—C19	120.9 (2)	H511—C51—H512	107.8
C13—C18—C19	117.9 (2)	C47—C52—C51	112.5 (5)
C18—C19—C20	115.1 (2)	C47—C52—H521	109.1
C18—C19—H191	108.5	C51—C52—H521	109.1
C20—C19—H191	108.5	C47—C52—H522	109.1
C18—C19—H192	108.5	C51—C52—H522	109.1
C20—C19—H192	108.5	H521—C52—H522	107.8
H191—C19—H192	107.5	C34—C53—H531	109.5
C21—C20—C29	109.6 (2)	C34—C53—H532	109.5
C21—C20—C19	108.1 (2)	H531—C53—H532	109.5
C29—C20—C19	109.6 (2)	C34—C53—H533	109.5
C21—C20—C30	110.8 (2)	H531—C53—H533	109.5
C29—C20—C30	108.9 (2)	H532—C53—H533	109.5
C19—C20—C30	109.7 (2)	C34—C54—H541	109.5
C20—C21—C22	112.2 (2)	C34—C54—H542	109.5
C20—C21—H211	109.2	H541—C54—H542	109.5
C22—C21—H211	109.2	C34—C54—H543	109.5
C20—C21—H212	109.2	H541—C54—H543	109.5
C22—C21—H212	109.2	H542—C54—H543	109.5
H211—C21—H212	107.9	C40—C55—H551	109.5
C17—C22—C21	113.2 (2)	C40—C55—H552	109.5
C17—C22—H221	108.9	H551—C55—H552	109.5
C21—C22—H221	108.9	C40—C55—H553	109.5
C17—C22—H222	108.9	H551—C55—H553	109.5
C21—C22—H222	108.9	H552—C55—H553	109.5
H221—C22—H222	107.8	C38—C56—H561	109.5
C4—C23—H231	109.5	C38—C56—H562	109.5
C4—C23—H232	109.5	H561—C56—H562	109.5
H231—C23—H232	109.5	C38—C56—H563	109.5
C4—C23—H233	109.5	H561—C56—H563	109.5
H231—C23—H233	109.5	H562—C56—H563	109.5
H232—C23—H233	109.5	C44—C57—H571	109.5
C4—C24—H241	109.5	C44—C57—H572	109.5
C4—C24—H242	109.5	H571—C57—H572	109.5
H241—C24—H242	109.5	C44—C57—H573	109.5
C4—C24—H243	109.5	H571—C57—H573	109.5
H241—C24—H243	109.5	H572—C57—H573	109.5
H242—C24—H243	109.5	C76—C75—C44	107 (2)
C10—C25—H251	109.5	C76—C75—H751	110.3
C10—C25—H252	109.5	C44—C75—H751	110.3
H251—C25—H252	109.5	C76—C75—H752	110.3
C10—C25—H253	109.5	C44—C75—H752	110.3
H251—C25—H253	109.5	H751—C75—H752	108.6
H252—C25—H253	109.5	C75—C76—C77	124 (2)
C8—C26—H261	109.5	C75—C76—H761	106.3
C8—C26—H262	109.5	C77—C76—H761	106.3
H261—C26—H262	109.5	C75—C76—H762	106.3
C8—C26—H263	109.5	C77—C76—H762	106.3
H261—C26—H263	109.5	H761—C76—H762	106.4
H262—C26—H263	109.5	C78—C77—C82	121.8 (10)
C14—C27—H271	109.5	C78—C77—C76	119.0 (15)
C14—C27—H272	109.5	C82—C77—C76	118.7 (14)
H271—C27—H272	109.5	C77—C78—C43	120.4 (11)
C14—C27—H273	109.5	C77—C78—C79	121.5 (11)
H271—C27—H273	109.5	C43—C78—C79	117.4 (9)
H272—C27—H273	109.5	C80—C79—C78	113.4 (9)
C20—C29—H291	109.5	C80—C79—H791	108.9
C20—C29—H292	109.5	C78—C79—H791	108.9
H291—C29—H292	109.5	C80—C79—H792	108.9
C20—C29—H293	109.5	C78—C79—H792	108.9
H291—C29—H293	109.5	H791—C79—H792	107.7
H292—C29—H293	109.5	C79—C80—C81	108.9 (16)
C20—C30—H301	109.5	C79—C80—C90	110 (3)
C20—C30—H302	109.5	C81—C80—C90	112 (3)
H301—C30—H302	109.5	C79—C80—C89	109 (3)
C20—C30—H303	109.5	C81—C80—C89	109 (3)
H301—C30—H303	109.5	C90—C80—C89	107.9 (16)
H302—C30—H303	109.5	C82—C81—C80	110.6 (12)
C32—C31—C40	112.41 (18)	C82—C81—H811	109.5
C32—C31—H311	109.1	C80—C81—H811	109.5
C40—C31—H311	109.1	C82—C81—H812	109.5
C32—C31—H312	109.1	C80—C81—H812	109.5
C40—C31—H312	109.1	H811—C81—H812	108.1
H311—C31—H312	107.9	C81—C82—C77	114.7 (7)
C33—C32—C31	112.04 (19)	C81—C82—H821	108.6
C33—C32—H321	109.2	C77—C82—H821	108.6
C31—C32—H321	109.2	C81—C82—H822	108.6
C33—C32—H322	109.2	C77—C82—H822	108.6
C31—C32—H322	109.2	H821—C82—H822	107.6
H321—C32—H322	107.9	C80—C89—H891	109.5
O33—C33—C32	111.34 (19)	C80—C89—H892	109.5
O33—C33—C34	113.27 (19)	H891—C89—H892	109.5
C32—C33—C34	113.07 (19)	C80—C89—H893	109.5
O33—C33—H331	106.2	H891—C89—H893	109.5
C32—C33—H331	106.2	H892—C89—H893	109.5
C34—C33—H331	106.2	C80—C90—H901	109.5
C33—O33—H33	112 (3)	C80—C90—H902	109.5
C54—C34—C53	107.3 (2)	H901—C90—H902	109.5
C54—C34—C33	110.85 (19)	C80—C90—H903	109.5
C53—C34—C33	107.43 (19)	H901—C90—H903	109.5
C54—C34—C35	114.02 (19)	H902—C90—H903	109.5
C53—C34—C35	109.02 (18)		
			
C10—C1—C2—C3	−57.5 (3)	C37—C38—C39—C41	179.90 (18)
C1—C2—C3—O3	175.5 (2)	C56—C38—C39—C41	61.0 (2)
C1—C2—C3—C4	54.7 (3)	C44—C38—C39—C41	−60.2 (2)
O3—C3—C4—C24	−48.6 (3)	C37—C38—C39—C40	46.6 (2)
C2—C3—C4—C24	75.4 (3)	C56—C38—C39—C40	−72.3 (2)
O3—C3—C4—C23	68.0 (2)	C44—C38—C39—C40	166.49 (18)
C2—C3—C4—C23	−168.1 (2)	C32—C31—C40—C55	−68.7 (2)
O3—C3—C4—C5	−174.30 (19)	C32—C31—C40—C35	53.9 (2)
C2—C3—C4—C5	−50.3 (3)	C32—C31—C40—C39	169.01 (18)
C3—C4—C5—C6	−176.4 (2)	C36—C35—C40—C31	173.24 (18)
C24—C4—C5—C6	59.5 (3)	C34—C35—C40—C31	−53.5 (2)
C23—C4—C5—C6	−60.1 (3)	C36—C35—C40—C55	−66.8 (2)
C3—C4—C5—C10	53.1 (3)	C34—C35—C40—C55	66.5 (2)
C24—C4—C5—C10	−71.0 (3)	C36—C35—C40—C39	57.2 (2)
C23—C4—C5—C10	169.4 (2)	C34—C35—C40—C39	−169.58 (18)
C10—C5—C6—C7	−65.1 (2)	C41—C39—C40—C31	64.2 (2)
C4—C5—C6—C7	161.72 (19)	C38—C39—C40—C31	−165.71 (18)
C5—C6—C7—C8	60.4 (3)	C41—C39—C40—C55	−56.0 (2)
C6—C7—C8—C26	71.7 (2)	C38—C39—C40—C55	74.0 (2)
C6—C7—C8—C9	−49.2 (3)	C41—C39—C40—C35	179.43 (18)
C6—C7—C8—C14	−168.62 (18)	C38—C39—C40—C35	−50.5 (2)
C26—C8—C9—C11	61.2 (2)	C40—C39—C41—O41	−5.8 (3)
C7—C8—C9—C11	−179.55 (19)	C38—C39—C41—O41	−140.4 (2)
C14—C8—C9—C11	−59.7 (2)	C40—C39—C41—C42	175.1 (2)
C26—C8—C9—C10	−73.1 (2)	C38—C39—C41—C42	40.5 (3)
C7—C8—C9—C10	46.2 (3)	O41—C41—C42—C43	170.1 (3)
C14—C8—C9—C10	166.01 (18)	C39—C41—C42—C43	−10.8 (4)
C2—C1—C10—C25	−68.2 (2)	C41—C42—C43—C48	−171.0 (4)
C2—C1—C10—C5	55.5 (2)	C41—C42—C43—C78	167.0 (4)
C2—C1—C10—C9	169.54 (18)	C41—C42—C43—C44	0.4 (5)
C6—C5—C10—C25	−66.5 (2)	C42—C43—C44—C45	−142.6 (10)
C4—C5—C10—C25	65.1 (3)	C48—C43—C44—C45	28.8 (11)
C6—C5—C10—C1	172.94 (18)	C78—C43—C44—C45	50.0 (11)
C4—C5—C10—C1	−55.4 (2)	C42—C43—C44—C57	101.6 (3)
C6—C5—C10—C9	57.8 (2)	C48—C43—C44—C57	−87.0 (4)
C4—C5—C10—C9	−170.58 (18)	C78—C43—C44—C57	−65.8 (4)
C11—C9—C10—C25	−55.8 (2)	C42—C43—C44—C75	−141.9 (14)
C8—C9—C10—C25	75.2 (2)	C48—C43—C44—C75	29.5 (14)
C11—C9—C10—C1	64.7 (2)	C78—C43—C44—C75	50.7 (14)
C8—C9—C10—C1	−164.32 (19)	C42—C43—C44—C38	−21.3 (4)
C11—C9—C10—C5	178.69 (18)	C48—C43—C44—C38	150.1 (4)
C8—C9—C10—C5	−50.3 (2)	C78—C43—C44—C38	171.3 (4)
C10—C9—C11—O11	−8.0 (3)	C37—C38—C44—C43	170.5 (2)
C8—C9—C11—O11	−143.4 (2)	C56—C38—C44—C43	−70.4 (3)
C10—C9—C11—C12	172.83 (19)	C39—C38—C44—C43	51.1 (3)
C8—C9—C11—C12	37.4 (3)	C37—C38—C44—C45	−74.0 (10)
O11—C11—C12—C13	171.2 (2)	C56—C38—C44—C45	45.2 (10)
C9—C11—C12—C13	−9.5 (3)	C39—C38—C44—C45	166.6 (10)
C11—C12—C13—C18	−179.0 (2)	C37—C38—C44—C57	50.7 (3)
C11—C12—C13—C14	4.0 (3)	C56—C38—C44—C57	169.9 (2)
C12—C13—C14—C15	−148.8 (2)	C39—C38—C44—C57	−68.7 (2)
C18—C13—C14—C15	34.2 (3)	C37—C38—C44—C75	−65.8 (13)
C12—C13—C14—C27	95.4 (2)	C56—C38—C44—C75	53.4 (13)
C18—C13—C14—C27	−81.6 (2)	C39—C38—C44—C75	174.8 (13)
C12—C13—C14—C8	−27.2 (3)	C43—C44—C45—C46	−65.2 (17)
C18—C13—C14—C8	155.81 (19)	C57—C44—C45—C46	48.4 (18)
C26—C8—C14—C13	−66.9 (2)	C75—C44—C45—C46	119 (14)
C7—C8—C14—C13	174.14 (18)	C38—C44—C45—C46	174.4 (13)
C9—C8—C14—C13	54.8 (2)	C44—C45—C46—C47	69 (2)
C26—C8—C14—C15	52.4 (2)	C45—C46—C47—C48	−39 (2)
C7—C8—C14—C15	−66.6 (2)	C45—C46—C47—C52	161.2 (15)
C9—C8—C14—C15	174.10 (19)	C52—C47—C48—C49	−9.7 (10)
C26—C8—C14—C27	173.62 (18)	C46—C47—C48—C49	−167.9 (12)
C7—C8—C14—C27	54.6 (2)	C52—C47—C48—C43	170.2 (6)
C9—C8—C14—C27	−64.7 (2)	C46—C47—C48—C43	12.0 (14)
C13—C14—C15—C16	−58.5 (3)	C42—C43—C48—C47	165.3 (5)
C27—C14—C15—C16	57.0 (3)	C78—C43—C48—C47	−91 (3)
C8—C14—C15—C16	−179.2 (2)	C44—C43—C48—C47	−6.2 (8)
C14—C15—C16—C17	51.7 (3)	C42—C43—C48—C49	−14.7 (7)
C15—C16—C17—C18	−18.2 (3)	C78—C43—C48—C49	89 (3)
C15—C16—C17—C22	160.9 (2)	C44—C43—C48—C49	173.7 (4)
C16—C17—C18—C13	−6.6 (4)	C47—C48—C49—C50	21.1 (10)
C22—C17—C18—C13	174.3 (2)	C43—C48—C49—C50	−158.9 (7)
C16—C17—C18—C19	174.9 (2)	C48—C49—C50—C51	−44.0 (7)
C22—C17—C18—C19	−4.2 (4)	C48—C49—C50—C59	76.5 (12)
C12—C13—C18—C17	−179.5 (2)	C48—C49—C50—C60	−161.8 (9)
C14—C13—C18—C17	−2.5 (3)	C59—C50—C51—C52	−62 (3)
C12—C13—C18—C19	−0.9 (3)	C60—C50—C51—C52	175.8 (16)
C14—C13—C18—C19	176.1 (2)	C49—C50—C51—C52	57.9 (9)
C17—C18—C19—C20	−14.4 (3)	C48—C47—C52—C51	22.6 (10)
C13—C18—C19—C20	167.0 (2)	C46—C47—C52—C51	−177.1 (12)
C18—C19—C20—C21	45.4 (3)	C50—C51—C52—C47	−47.7 (18)
C18—C19—C20—C29	164.8 (2)	C43—C44—C75—C76	−48 (2)
C18—C19—C20—C30	−75.6 (3)	C45—C44—C75—C76	−44 (12)
C29—C20—C21—C22	−179.7 (2)	C57—C44—C75—C76	69 (2)
C19—C20—C21—C22	−60.3 (3)	C38—C44—C75—C76	−171.1 (18)
C30—C20—C21—C22	60.0 (3)	C44—C75—C76—C77	24 (4)
C18—C17—C22—C21	−10.5 (4)	C75—C76—C77—C78	3 (4)
C16—C17—C22—C21	170.4 (2)	C75—C76—C77—C82	175 (3)
C20—C21—C22—C17	43.6 (3)	C82—C77—C78—C43	−176.6 (7)
C40—C31—C32—C33	−58.0 (3)	C76—C77—C78—C43	−4.3 (18)
C31—C32—C33—O33	−174.44 (19)	C82—C77—C78—C79	−6.3 (12)
C31—C32—C33—C34	56.7 (3)	C76—C77—C78—C79	166.0 (16)
O33—C33—C34—C54	−53.9 (3)	C42—C43—C78—C77	170.2 (6)
C32—C33—C34—C54	74.0 (2)	C48—C43—C78—C77	82 (3)
O33—C33—C34—C53	63.1 (2)	C44—C43—C78—C77	−22.5 (8)
C32—C33—C34—C53	−169.1 (2)	C42—C43—C78—C79	−0.5 (8)
O33—C33—C34—C35	−179.47 (18)	C48—C43—C78—C79	−89 (3)
C32—C33—C34—C35	−51.6 (2)	C44—C43—C78—C79	166.8 (5)
C54—C34—C35—C36	60.6 (2)	C77—C78—C79—C80	−14.6 (12)
C53—C34—C35—C36	−59.3 (2)	C43—C78—C79—C80	156.0 (9)
C33—C34—C35—C36	−175.74 (18)	C78—C79—C80—C81	47.8 (8)
C54—C34—C35—C40	−71.3 (3)	C78—C79—C80—C90	−74.9 (15)
C53—C34—C35—C40	168.8 (2)	C78—C79—C80—C89	166.7 (10)
C33—C34—C35—C40	52.4 (2)	C79—C80—C81—C82	−62.1 (11)
C40—C35—C36—C37	−63.8 (2)	C90—C80—C81—C82	60 (4)
C34—C35—C36—C37	161.52 (18)	C89—C80—C81—C82	179 (2)
C35—C36—C37—C38	59.8 (3)	C80—C81—C82—C77	43 (2)
C36—C37—C38—C56	70.9 (2)	C78—C77—C82—C81	−8.2 (12)
C36—C37—C38—C39	−49.3 (3)	C76—C77—C82—C81	179.5 (16)
C36—C37—C38—C44	−168.9 (2)		

### Hydrogen-bond geometry (Å, º)

**Table:**

*D*—H···*A*	*D*—H	H···*A*	*D*···*A*	*D*—H···*A*
O3—H3···O11^i^	0.84	2.04 (2)	2.792 (2)	148 (4)
O33—H33···O41^ii^	0.84	2.13 (2)	2.921 (2)	158 (4)

Symmetry codes: (i) −*x*+1, *y*−1/2, −*z*+2; (ii) −*x*+1, *y*+1/2, −*z*+1.
